# Supplementary material for: Thermodynamic analog of integrate-and-fire neuronal networks by maximum entropy modelling
Source: Sci Rep. 2024 Apr 25;14:9480. doi: 10.1038/s41598-024-60117-3 (PMC11045794; doi:10.1038/s41598-024-60117-3)
Supplement: Supplementary file 1 — Supplementary Information. [file 41598_2024_60117_MOESM1_ESM.pdf]

Supplementary Information

Thermodynamic analog of integrate-and-fire neuronal networks by maximum entropy modelling

T. S. A. N. Simões,[1] C. I. N. Sampaio Filho,[2] H. J. Herrmann,[2,3] J.S. Andrade Jr.,[2] and L. de Arcangelis[1]

[1] *Department of Mathematics and Physics, University of Campania "Luigi Vanvitelli", Viale Lincoln, 5, 81100 Caserta, Italy*
[2] *Departamento de Física, Universidade Federal do Ceará, 60451-970, Fortaleza, Ceará, Brazil*
[3] *PMMH, ESPCI, 7 quai St. Bernard, 75005 Paris, France*

(Dated: January 29, 2024)

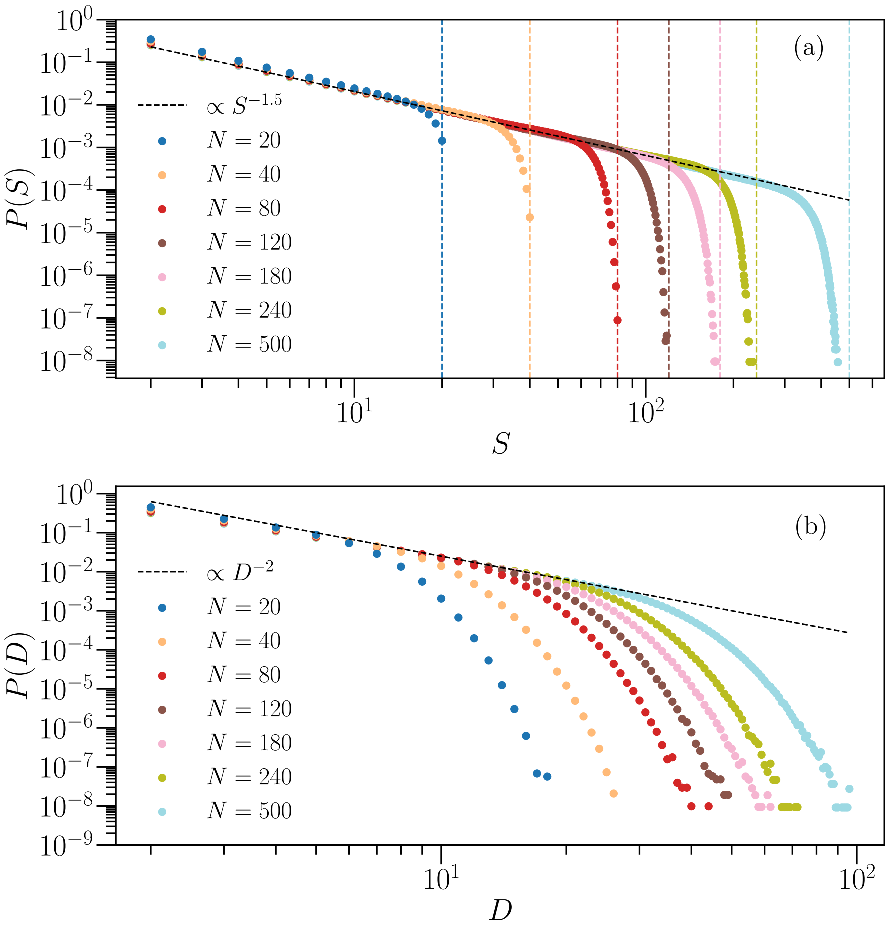

FIG. S1. **Avalanche size and duration distributions of critical fully-excitatory IF networks.** Avalanche size S (a) and duration D (b) distributions for several system sizes N tuned to the critical state. The values of δu_{rec} for each N are reported in table (S1). Results are averages over $2 \cdot 10^8$ avalanches, generated over $N_c = 10^4$ different network configurations. The vertical dashed lines in (a) indicate the system size of the corresponding colour-matching curve of $P(S)$.

TABLE S1. Values of δu_{rec} for the fully-excitatory systems considered in Fig. (S1)

**System size** N	$\delta u_{\mathbf{rec}}$
20	0.00190
40	0.00150
80	0.00120
120	0.00100
180	0.00080
240	0.00075
500	0.00050

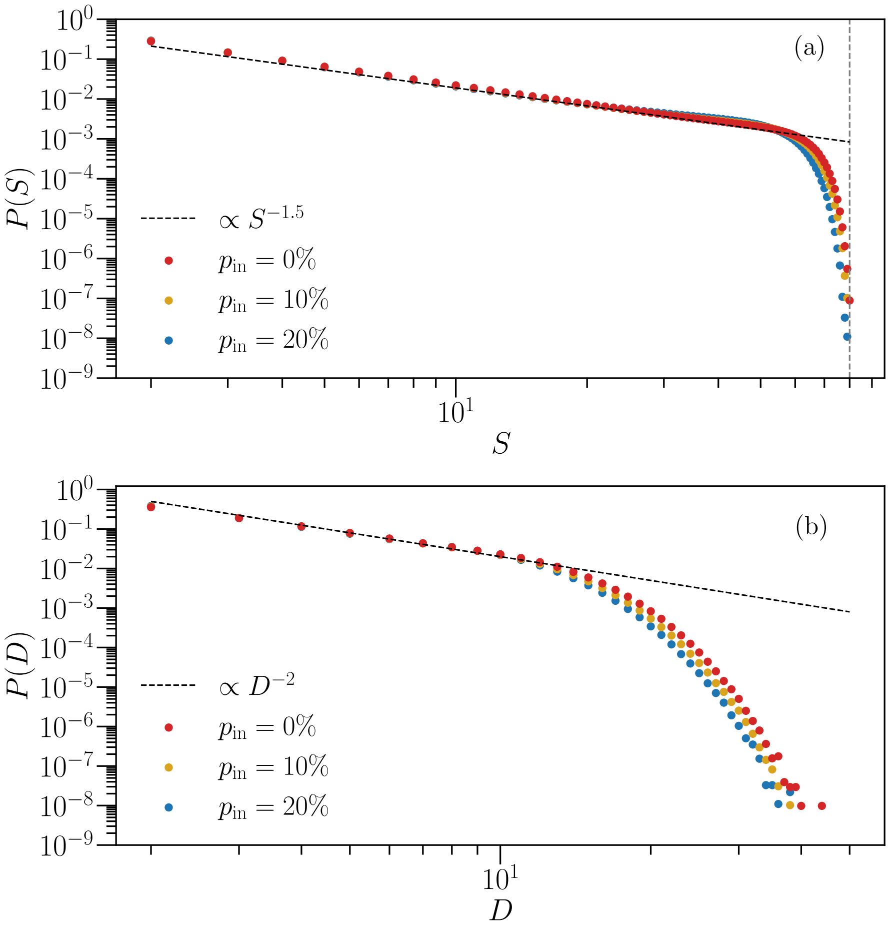

FIG. S2. **Avalanche size and duration distributions of critical IF networks with different fractions of inhibitory neurons.** Avalanche size S (a) and duration D (b) distributions for systems with $N = 80$ and different percentage p_{in} of inhibitory neurons, tuned to the critical state. The values of δu_{rec} for each p_{in} are reported in table (S2). Results are averages over $2 \cdot 10^8$ avalanches, generated over $N_c = 10^4$ different network configurations. The vertical dashed line in (a) indicates the system size $N = 80$.

TABLE S2. Values of δu_{rec} for the systems with $N = 80$ considered in Fig. (S2)

$p_{\mathbf{in}}$	$\delta u_{\mathbf{rec}}$
0%	0.0012
10%	0.0014
20%	0.0017

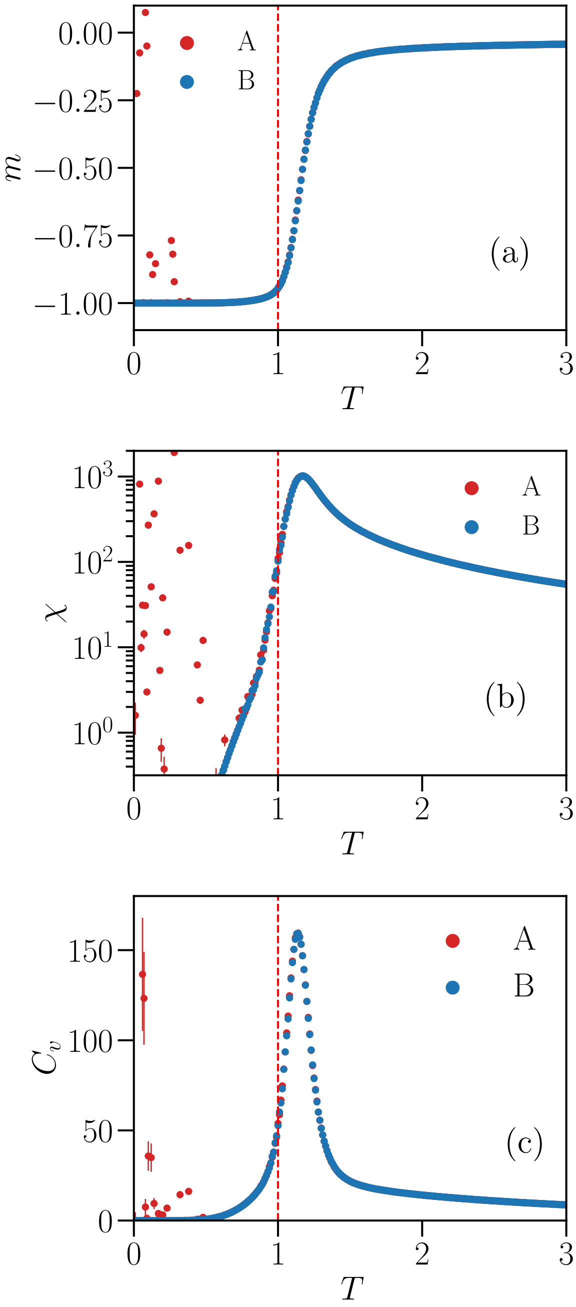

FIG. S3. **Dependence with the initial spin configuration of the thermodynamic functions of Ising-like models associated with fully-excitatory IF networks.** Magnetization per spin m (a), susceptibility χ (b) and specific heat C_v (c) as a function of the temperature T of a pairwise Ising system associated to a network with $N = 80$ neurons, using two different initial spin configurations for MC sampling: starting with random σ_i (A) or with all $\sigma_i = -1$ (B). Results are averages over $M_c = 3 \cdot 10^6$ spin configurations. Error bars are given by the standard error.

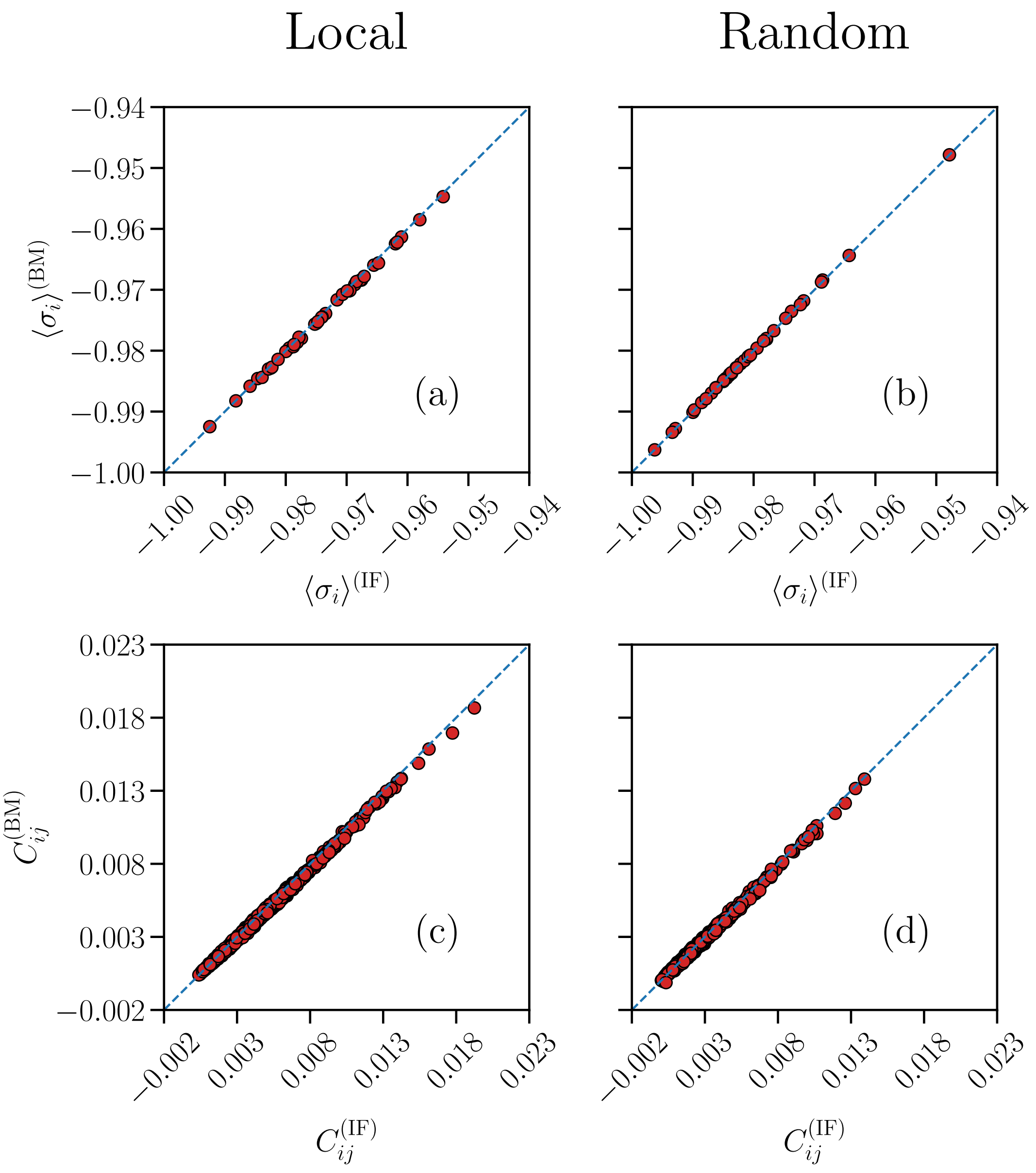

FIG. S4. **Quality test for the BM learning process for IF subnetworks.** Comparison between the average local activity $\langle\sigma_i\rangle$ (a-b) and correlation functions C_{ij} (c-d) of the pairwise Ising model (y-axes) and IF model network (x-axes), for subnetworks with $n = 40$ neurons, with a local and random spatial distribution, in a system with $N = 500$ neurons. The blue dashed lines are given by the bisector $y = x$. Results are averages over $N_b = 10^7$ time bins for the IF model (IF), and averages over $M_c = 3 \cdot 10^6$ spin configurations for the Ising model (BM). Error bars are given by the standard error, and are smaller or equal to the symbol size.

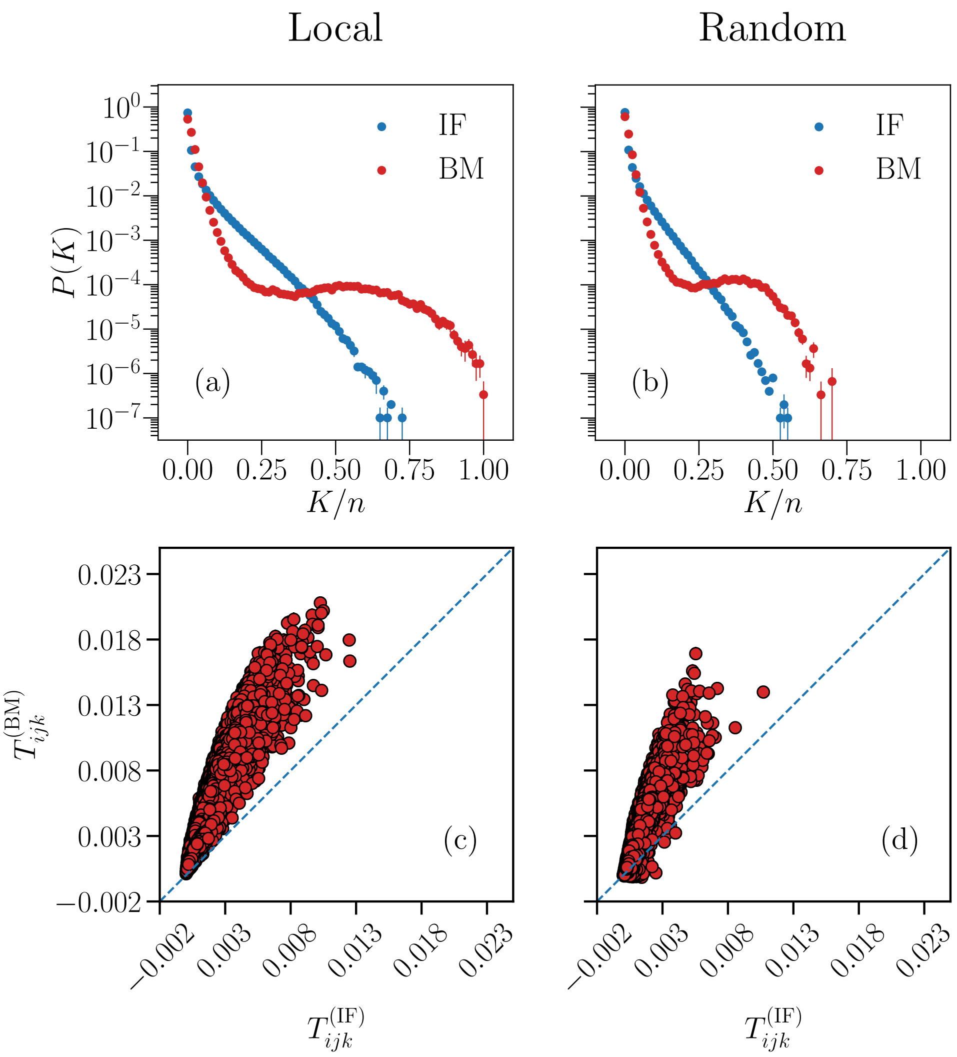

FIG. S5. **Predictive capability of the Ising model for IF model subnetworks $n = 80$ in a system of size $N = 500$.** Comparison between Monte Carlo sampling of the Ising model (BM) and the neural network data (IF) of the probability $P(K)$ (a-b) and triplets T_{ijk} (c-d), for subnetworks with $n = 80$ neurons, with a local (a and c) and random (b and d) spatial distribution, in a system with $N = 500$ neurons. The blue dashed lines in the bottom plots are the bisector $y = x$. Results are averages over $N_b = 10^7$ time bins for the IF model (IF), and averages over $M_c = 3 \cdot 10^6$ spin configurations for the Ising model (BM). Error bars are given by the standard error, and are overall smaller or equal to the symbol size.

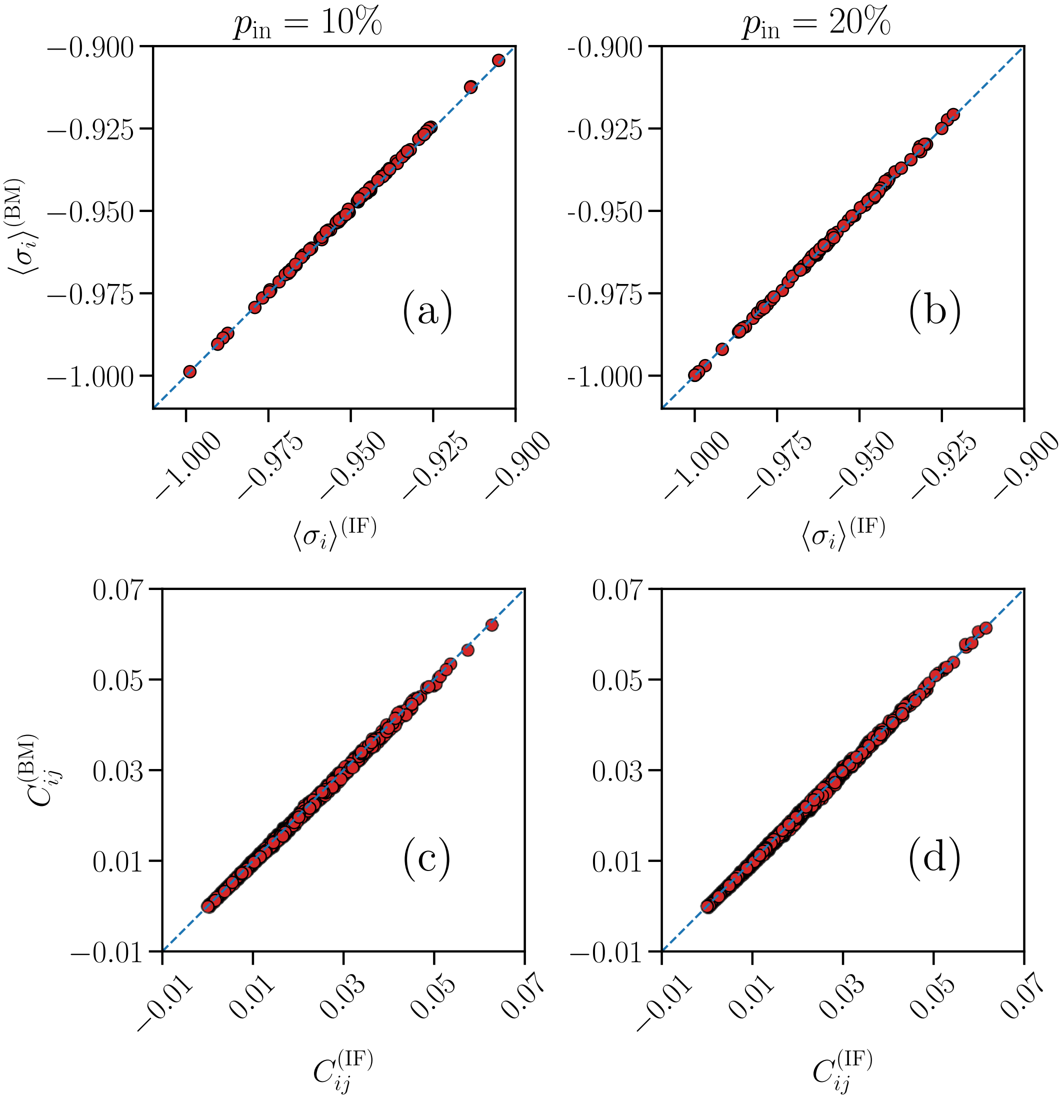

FIG. S6. **Quality test for the BM learning process for IF subnetworks with different fractions of inhibitory neurons.** Comparison between the average local activity $\langle\sigma_i\rangle$ (a-b) and correlation functions C_{ij} (c-d) of the pairwise Ising model (y-axes) and IF model network (x-axes), for a IF network of size $N = 80$ with different fractions $p_{\rm in} = \{10\%, 20\%\}$ of inhibitory neurons. The blue dashed lines are given by the bisector $y = x$. Results are averages over $N_b = 10^7$ time bins for the IF model (IF), and averages over $M_c = 3 \cdot 10^6$ spin configurations for the Ising model (BM). Error bars are given by the standard error, and are smaller or equal to the symbol size.

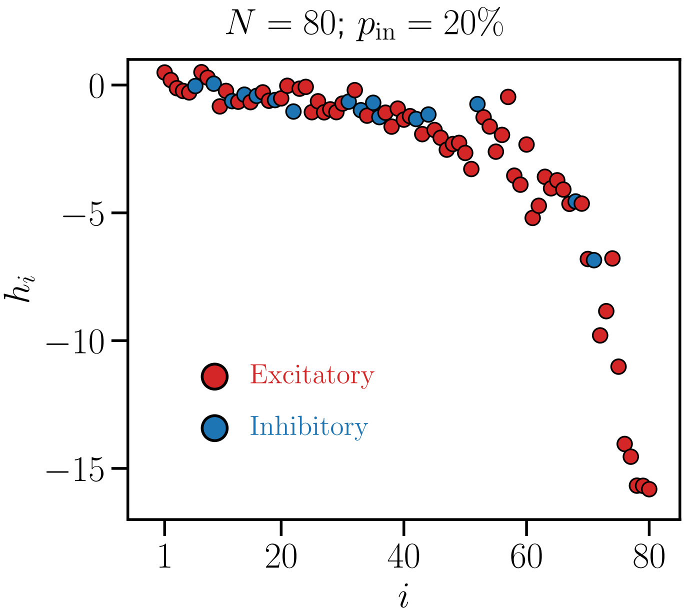

FIG. S7. **Learned fields h_i of the pairwise Ising model associated with an IF network with inhibitory neurons.** Plot of the fields h_i, sorted by the average local activity $\langle \sigma_i \rangle^{(\rm IF)}$ of the associated neuron i, in order of decreasing $\langle \sigma_i \rangle^{(\rm IF)}$, for a IF network with $N = 80$ and $p_{\rm in} = 20\%$ inhibitory neurons (same data of the distribution presented in Fig. (11)a in the main text, for the same $p_{\rm in}$). Red symbols indicate fields h_i associated with an excitatory neuron, while blue symbols correspond to h_i associated with inhibitory ones.

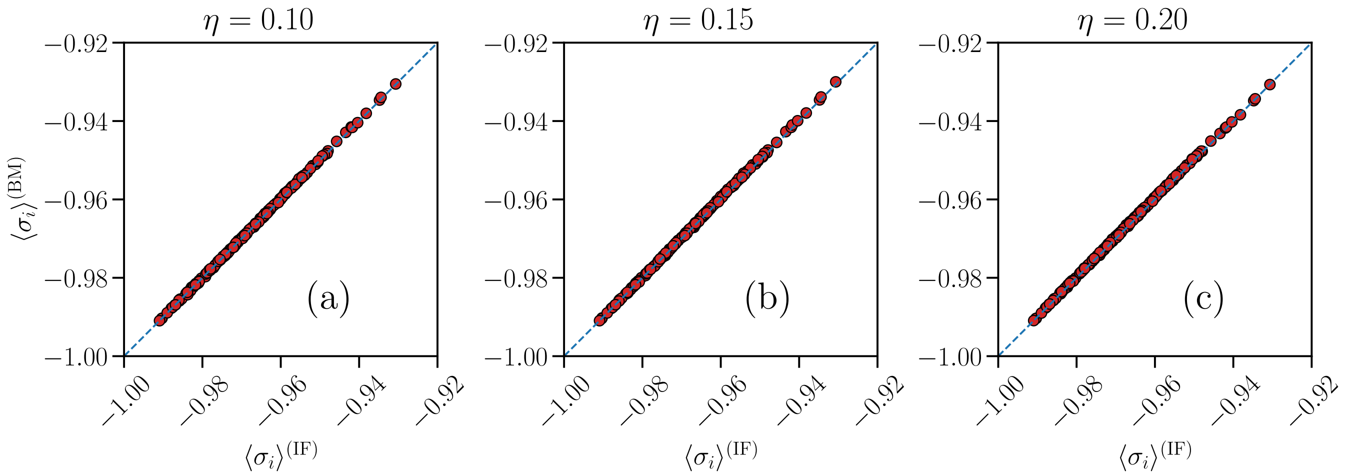

FIG. S8. **Quality test for the BM learning process with a partially-connected Ising model of the average local activities.** Comparison between the average local activity $\langle \sigma_i \rangle$ of the partially-connected pairwise Ising model (y-axes) and IF model network (x-axes), for a fully-excitatory system of size $N = 180$, considering three different thresholds $\eta = \{0.10, 0.15, 0.20\}$ for the removal of a subset of the J_{ij} (see main text). The blue dashed lines are given by the bisector $y = x$. Results are averages over $N_b = 10^7$ time bins for the IF model (IF), and averages over $M_c = 3 \cdot 10^7$ spin configurations for the Ising model (BM). Error bars are given by the standard error, and are smaller or equal to the symbol size.

DERIVATION OF THE MAXIMUM ENTROPY PROBABILITY DISTRIBUTION

Consider a random variable $\boldsymbol{\sigma}$ defined by a probability distribution $P(\boldsymbol{\sigma})$ which is unknown *a priori*. This distribution $P(\boldsymbol{\sigma})$ must, of course, obey the normalization condition,

$$\sum_{\boldsymbol{\sigma}} P(\boldsymbol{\sigma}) = 1 \ , \tag{1}$$

where $\sum_{\boldsymbol{\sigma}}$ indicates a sum over all possible outcomes of $\boldsymbol{\sigma}$. We want to find an expression for $P(\boldsymbol{\sigma})$ given the average value of some function $f(\boldsymbol{\sigma})$ of the random variable $\boldsymbol{\sigma}$,

$$\langle f \rangle = \sum_{\boldsymbol{\sigma}} P(\boldsymbol{\sigma}) f(\boldsymbol{\sigma}) \ . \tag{2}$$

The maximum entropy principle [1] states that the best choice for $P(\boldsymbol{\sigma})$ out of all possible distributions which are consistent with previous knowledge (in this case, the average $\langle f \rangle$) is the one that has the maximum entropy, i.e. the least biased one. In other words, we should use the probability distribution that maximizes the entropy [2],

$$\mathcal{S} = -a \sum_{\boldsymbol{\sigma}} P(\boldsymbol{\sigma}) \ln[P(\boldsymbol{\sigma})] \ , \tag{3}$$

while subject to constraints (1) and (2), where a is a positive constant that defines the units of $\mathcal{S}$, which we set to $a = 1$ without loss of generality. To solve this problem, we can make use of the method of Lagrangian multipliers [1]. For Lagrangian multipliers λ_0 and λ_1 associated with the constraints (1) and (2), respectively, the Lagrangian reads,

$$\mathcal{L}[P(\boldsymbol{\sigma})] = -\sum_{\boldsymbol{\sigma}} P(\boldsymbol{\sigma}) \ln[P(\boldsymbol{\sigma})] + \lambda_0 \cdot \left(\sum_{\boldsymbol{\sigma}} P(\boldsymbol{\sigma}) - 1 \right) + \lambda_1 \cdot \left(\sum_{\boldsymbol{\sigma}} P(\boldsymbol{\sigma}) f(\boldsymbol{\sigma}) - \langle f \rangle \right) \ . \tag{4}$$

To proceed, it is useful to explicitly define each probability $p_i \equiv P(\boldsymbol{\sigma} = \boldsymbol{\sigma}^i)$, where $\boldsymbol{\sigma}^i$ is a particular outcome of the random variable $\boldsymbol{\sigma}$. Since the Lagrangian (4) depends on a function, $P(\boldsymbol{\sigma})$, $\mathcal{L}$ is a functional, and to search for a local extremum with respect to $P(\boldsymbol{\sigma})$ we require the following functional (or variational) derivative [3],

$$\begin{aligned} \frac{\delta \mathcal{L}}{\delta P(\boldsymbol{\sigma})}(\boldsymbol{\sigma}^i) &\equiv \lim_{\varepsilon \to 0} \frac{1}{\varepsilon} \cdot \left(\mathcal{L}[P(\boldsymbol{\sigma}) + \varepsilon \delta_{\boldsymbol{\sigma},\boldsymbol{\sigma}^i}] - \mathcal{L}[P(\boldsymbol{\sigma})] \right) = \\ &= \frac{d\mathcal{L}}{dP(\boldsymbol{\sigma})} \delta_{\boldsymbol{\sigma},\boldsymbol{\sigma}^i} \ , \end{aligned} \tag{5}$$

where ε is a real number and $\delta_{\boldsymbol{\sigma},\boldsymbol{\sigma}^i}$ is the delta Kronecker function. The equality follows from multiplying and dividing the right side by $\delta_{\boldsymbol{\sigma},\boldsymbol{\sigma}^i}$ and then taking the limit $\varepsilon \to 0$. Equation (5) expresses the change in the functional $\mathcal{L}$ due to changes in the function $P(\boldsymbol{\sigma})$ at the particular point $\boldsymbol{\sigma} = \boldsymbol{\sigma}^i$. The extrema condition equation then implies

$$\begin{aligned} & \frac{d\mathcal{L}}{dP(\boldsymbol{\sigma})} \delta_{\boldsymbol{\sigma},\boldsymbol{\sigma}^i} = 0 \ , \\ \implies & -(\ln[p_i] + 1) + \lambda_0 + \lambda_1 f(\boldsymbol{\sigma}^i) = 0 \ , \\ \implies & p_i = p_0 e^{\lambda_1 f(\boldsymbol{\sigma}^i)} \end{aligned} \tag{6}$$

with $p_0 \equiv e^{1+\lambda_0}$. Since the last result is identical for all other probabilities $p_{j \neq i}$, we have for the probability distribution $P(\boldsymbol{\sigma})$,

$$P(\boldsymbol{\sigma}) = p_0 e^{\lambda_1 f(\boldsymbol{\sigma})} \ . \tag{7}$$

The factor p_0 is determined by the normalization condition (1),

$$p_0 = \frac{1}{\sum_{\boldsymbol{\sigma}} e^{\lambda_1 f(\boldsymbol{\sigma})}} \equiv \frac{1}{Z} \ , \tag{8}$$

so

$$P(\boldsymbol{\sigma}) = \frac{e^{\lambda_1 f(\boldsymbol{\sigma})}}{Z} \ . \tag{9}$$

The last result can be easily generalized for the case that we have instead C constraints associated with the average value of C functions of the random variable $\boldsymbol{\sigma}$, $\{f_1(\boldsymbol{\sigma}), f_2(\boldsymbol{\sigma}), ..., f_C(\boldsymbol{\sigma})\}$. In this case, besides λ_0, associated with the normalization condition, one has C Lagrangian multipliers $\{\lambda_1, \lambda_2, ..., \lambda_C\}$, and the last term in the Lagrangian (4) needs to be modified by including a sum over the C constraints. This then gives

$$P(\boldsymbol{\sigma}) = \frac{1}{Z} e^{\sum_{c=1}^{C} \lambda_c f_c(\boldsymbol{\sigma})} \ , \tag{10}$$

$$Z = \sum_{\boldsymbol{\sigma}} e^{\sum_{c=1}^{C} \lambda_c f_c(\boldsymbol{\sigma})} \ . \tag{11}$$

Going back now to the particular case of the IF model, for a given network with N neurons, as a first approximation, we can aim at constraining the single-neuron $\langle\sigma_i\rangle$ and pairwise $\langle\sigma_i\sigma_j\rangle$ information, and, by extension, the correlation functions C_{ij} (see "Firing statistics" in the Results section). This corresponds to $N + N\cdot(N-1)/2$ constraints, giving N Lagrangian multipliers associated with each $f_i(\boldsymbol{\sigma}) = \sigma_i$, that we will denote as h_i, and $N\cdot(N-1)/2$ multipliers for each different pair $f_{ij}(\boldsymbol{\sigma}) = \sigma_i\sigma_j$, which we will denote in turn as J_{ij}. For this case, the maximum entropy probability (10) reads,

$$P(\boldsymbol{\sigma}) = \frac{1}{Z} e^{-H(\boldsymbol{\sigma})} \ , \tag{12}$$

$$Z = \sum_{\boldsymbol{\sigma}} e^{-H(\boldsymbol{\sigma})} \ , \tag{13}$$

$$H(\boldsymbol{\sigma}) = -\sum_{i}^{N} h_i\sigma_i - \sum_{i}^{N}\sum_{j<i}^{N} J_{ij}\sigma_i\sigma_j \ . \tag{14}$$

Equations (12)-(14) are the expressions for the least-biased probability distribution that is consistent with the measured values of $\langle\sigma_i\rangle$ and C_{ij} in the IF model. This is the Boltzmann distribution, and is mathematically equivalent to the distribution of spin configurations of a generalized Ising model [4],

$$P_{\text{Ising}}(\boldsymbol{\sigma}) = \frac{1}{Z_{\text{Ising}}} e^{-\frac{H(\boldsymbol{\sigma})}{T}} \ , \tag{15}$$

$$Z_{\text{Ising}} = \sum_{\boldsymbol{\sigma}} e^{-\frac{H(\boldsymbol{\sigma})}{T}} \ , \tag{16}$$

with temperature $T = 1$ in natural units (Boltzmann constant $k_B = 1$), and where $H(\boldsymbol{\sigma})$ is the Hamiltonian or energy function and Z_{Ising} is the partition function, whose sum runs over all 2^N possible configurations of spin states $\boldsymbol{\sigma}$.

---

[1] E. T. Jaynes, Physical Review **106**, 62 (1957), publisher: American Physical Society.
[2] H. C. Nguyen, R. Zecchina, and J. Berg, Advances in Physics **66**, 197 (2017), publisher: Taylor & Francis _eprint: https://doi.org/10.1080/00018732.2017.1341604.
[3] I. M. Gelfand and S. V. Fomin, English*Calculus of Variations* (Dover Publications, New York, 2000).
[4] G. Tkacik, E. Schneidman, M. J. Berry II, and W. Bialek, arXiv:0912.5409 [q-bio] (2009), arXiv: 0912.5409.
